# Impact of initial autonomic dysfunction on long-term psychocognitive and functional prognoses in older patients with acute cerebral infarction

**DOI:** 10.3389/fnins.2026.1769836

**Published:** 2026-03-30

**Authors:** Guangzhou Yu, Ligong Gao, Hao Zhang, Shuli Dong, Yang Yang

**Affiliations:** 1 Department of Neurology, Zhumadian Central Hospital, Zhumadian, Henan, China; 2 Department of Neurosurgery, Zhumadian Central Hospital, Zhumadian, Henan, China

**Keywords:** acute cerebral infarction, autonomic dysfunction, cognitive function, functional ability, heart rate variability, posttraumatic growth

## Abstract

**Background:**

The prognostic value of autonomic dysfunction, an identifiable and reversible factor, in patients with cerebrovascular diseases has not been fully elucidated. This study aimed to explore the impact of baseline autonomic function on long-term psychocognitive and functional prognoses in older patients with acute cerebral infarction.

**Methods:**

A total of 420 older patients with acute cerebral infarction who received interventional therapy were enrolled. Baseline assessment was performed on days 7–10 after symptom onset. According to baseline heart rate variability (HRV), patients were classified into the HRV-normal group (*n* = 113) and the HRV-abnormal group (*n* = 307). All patients were followed up for 1 year. At baseline and at 1-year follow-up, six scales (including PTGI, CD-RISC, MoCA, ACE-III, MBI, and FIM) were used to evaluate posttraumatic growth, cognitive function, and functional ability. Multivariate logistic regression analysis was performed.

**Results:**

In the HRV-normal group, the probability of achieving favorable performance in all six scores at 1-year follow-up was significantly higher by 20.1 to 88.5% compared with baseline (all *p* < 0.001). In the HRV-abnormal group, the probability of favorable performance in these scores at 1-year follow-up did not increase relative to baseline (all *p* > 0.05). Furthermore, compared with the HRV-normal group, older patients with baseline autonomic abnormality had a 19.4 to 53.4% lower probability of achieving favorable psychocognitive and functional performance at 1-year follow-up (all *p* < 0.001).

**Conclusion:**

Initial autonomic dysfunction in older patients with acute cerebral infarction may adversely affect their long-term psychocognitive and functional prognoses.

## 1 Introduction

Each year, more than 12 million new cases of stroke occur worldwide, over 70% of which are ischemic strokes, with acute cerebral infarction (ACI) being the main type (Xia et al., 2024). In China, approximately 2 million new stroke cases are reported annually, and ACI accounts for a considerable proportion (Zhao et al., 2023). Individuals aged 60 years and older are at high risk of ACI, and the risk nearly doubles with every 10-year increase in age (Sharrief and Grotta, 2019). ACI is characterized by high mortality and high disability rates, and most survivors develop functional impairments that severely reduce quality of life.

As is well known, ACI can damage motor function in patients to varying degrees. With respect to cognitive and psychological outcomes, patients often experience cognitive decline after stroke, which may even progress to vascular dementia (Morgan and Mc Auley, 2024). They may also develop negative emotional disorders such as anxiety and depression (Gu et al., 2024). Meanwhile, patients may also exhibit positive psychological changes. Post-traumatic growth refers to improved psychological resilience and life perception in patients after experiencing disease-related trauma, which can provide important psychological support for rehabilitation and exert beneficial effects on long-term prognosis (Klass et al., 2025; Peng and Wan, 2018). Notably, post-traumatic growth and the maintenance of cognitive function may interact and reinforce each other (Gangstad et al., 2009; Calhoun et al., 2000). However, post-traumatic growth has not received sufficient attention in clinical practice.

Beyond the aforementioned effects on motor, cognitive, and psychological function, ACI frequently induces autonomic dysfunction, manifested mainly as imbalance between sympathetic and parasympathetic regulation (Barkhudaryan et al., 2025; Lago et al., 2025). Such imbalance can directly aggravate acute complications including cardiac arrhythmia. Meanwhile, the associations between autonomic function and psychological outcomes (e.g., post-traumatic growth) and cognitive function have drawn increasing attention. Several studies conducted among patients with major diseases, survivors of serious accidents, military personnel, and emergency responders have shown that better autonomic function is associated with higher post-traumatic growth (Karaman et al., 2025; Wei et al., 2017; Hourani et al., 2020), and it is also associated with superior cognitive performance under stress (Hourani et al., 2020). Although these studies are preliminary and cross-sectional, they suggest that the autonomic nervous system may be involved in the pathogenesis of cognitive and psychological changes after ACI and may even serve as a potential mediator in some pathways or mechanisms. Nevertheless, this hypothesis remains to be further verified.

In addition, heart rate variability (HRV) serves as a non-invasive, quantifiable, and relatively sensitive objective indicator for evaluating autonomic function. It can adequately reflect the regulatory balance and overall modulation capacity of the sympathetic and parasympathetic nerves, and has been widely used in studies on autonomic dysfunction in cerebrovascular diseases and the associations between autonomic function and cognition or psychology (Barkhudaryan et al., 2025; Lago et al., 2025; Karaman et al., 2025; Wei et al., 2017; Hourani et al., 2020).

Therefore, this study intends to adopt a prospective cohort design to systematically investigate the relationship between baseline autonomic abnormality and post-traumatic growth, cognitive function, and activities of daily living at 1-year follow-up in older patients with ACI. In this study, autonomic abnormality will be operationally defined and grouped based on HRV parameters, whereas autonomic dysfunction will be reserved for describing clinical manifestations, symptoms, and pathological conditions. This study is expected to preliminarily clarify the potential impact of HRV on key long-term rehabilitation indicators in this population, so as to provide a scientific basis for clinically evaluating rehabilitation potential and developing individualized intervention strategies.

## 2 Materials and methods

### 2.1 Ethical strategies

This study was approved by the Medical Ethics Committee of Zhumadian Central Hospital. All procedures were conducted in accordance with the requirements of the World Medical Association Declaration of Helsinki.

### 2.2 Participants

From January 1, 2020, to July 20, 2023, eligible participants were consecutively enrolled from older patients attending the Department of Neurology, Zhumadian Central Hospital, for inclusion in this study.

The inclusion criteria were as follows: (1) Aged ≥ 60 years; (2) Diagnosed with ACI in accordance with the World Health Organization diagnostic criteria for ischemic stroke, with confirmation by cranial computed tomography (cranial CT) (Mendelson and Prabhakaran, 2021); (3) Time from symptom onset to hospital admission ≤ 6 h; (4) National Institute of Health Stroke Scale (NIHSS) score ≤ 20; (5) All patients received comprehensive treatment centered on interventional therapy; (6) No history of cognitive impairment, dementia, or mental disorders; (7) No history of atrial fibrillation or third-degree atrioventricular block, with admission electrocardiogram results further confirming the absence of the aforementioned arrhythmias; (8) No history of autonomic nervous system-related diseases, trauma, or behaviors, including Parkinson’s disease, type 2 diabetes complicated with autonomic neuropathy, hyperthyroidism, hypothyroidism, long-term alcohol abuse (daily pure alcohol intake ≥ 20 g for a duration of ≥ 5 years), Guillain-Barré syndrome, and spinal cord injury; (9) No history of malignant tumors; (10) A history of common chronic diseases (e.g., hypertension, coronary heart disease, type 2 diabetes without autonomic neuropathy) was permitted, provided that the conditions were clinically stable; (11) No experience of other major traumatic events (e.g., the death of a family member, major surgery) within the past 12 months; (12) Willingness to participate in the study and provision of written informed consent.

Exclusion criteria were as follows: (1) Failure to meet the aforementioned inclusion criteria; (2) Missing key data in medical records; (3) Failure to complete all scheduled examinations and assessments in this study; (4) Failure to complete the 1-year follow-up; (5) Acute cerebrovascular disease recurs during the 1-year follow-up period.

### 2.3 Description of study period

In this study, the baseline was set at 10 to 14 days after the onset of ACI, and a 1-year follow-up was conducted starting from this time point. The baseline was chosen because older patients’ conditions had stabilized by then, which could reduce acute-phase interference and lower examination risks; a 1-year follow-up duration was sufficient to assess the long-term psychocognitive and functional prognoses.

### 2.4 Data collection

Basic characteristics of older patients at the onset of ACI and characteristics of ACI and acute-phase treatment in these patients were collected by reviewing medical records and interviewing family members. Basic characteristics of the patients included demographic data, personal history, disease history, and medication history. Characteristics of ACI and acute-phase treatment included pre-treatment status, intervention therapy details, and other therapies during hospitalization.

Smoking history was defined as regular smoking for ≥12 cumulative months in a lifetime, with ≥7 cigarettes per week. Drinking history was defined as regular alcohol consumption for ≥12 cumulative months in a lifetime, with ≥7 drinks per week. High-fat diet was defined as consuming fried foods, fatty meats, or animal offal on ≥5 days per week for the past 12 months, and using cooking oil in excess of typical amounts. High-salt diet was defined as consuming pickled or processed foods on ≥5 days per week for the past 12 months, and using salt in cooking in excess of typical amounts.

Disease history was confirmed based on diagnostic certificates or treatment records. Medication history referred to the medication use of patients in the 6 months prior to the onset of ACI.

Additionally, during the follow-up period, other cerebral infarction-related treatments were collected and documented using the aforementioned methods.

### 2.5 HRV assessment

At baseline, R-R interval data were collected from each patient for a minimum of 5 min using the Mindray BeneHeart R3a device (China). During this process, the device’s myoelectric and power frequency interference filtering functions were activated; patients remained in a prone resting position, avoided movement, and maintained a regular respiratory rhythm. Investigators monitored the screen in real time to ensure the rate of artifacts and arrhythmias was < 5% (i.e., < 15–25 abnormal intervals within 5 min).

After exporting the raw data, artifact correction was performed using the RHRV package in R software, and 256 consecutive abnormal-free R-R intervals were selected for analysis. This duration serves as an appropriate window for HRV assessment (Lombardi et al., 2018).

This study conducted HRV analysis from both time and frequency domains: In time-domain analysis, the standard deviation of normal-to-normal intervals (SDNN), root-mean square of successive differences between normal-to-normal intervals (RMSSD), and percentage of adjacent normal-to-normal intervals with a difference greater than 50 milliseconds (pNN50) were selected as core indicators. Among these, SDNN reflects the overall level of heart rate variability and demonstrates the coordinated regulatory ability of the sympathetic and parasympathetic nerves on the heart; RMSSD and pNN50 focus on short-term heart rate variability and specifically reflect the immediate regulatory activity of the parasympathetic nerve (vagus nerve). The combined use of these three indicators enables comprehensive assessment of autonomic nervous function. In frequency-domain analysis, low-frequency power (LF, 0.04–0.15 Hz), high-frequency power (HF, 0.15–0.40 Hz), and the LF/HF ratio were used as assessment indicators: LF is co-regulated by the sympathetic and parasympathetic nerves (with the sympathetic nerve as the dominant influence), HF is mainly regulated by the parasympathetic nerve, and the LF/HF ratio is used to quantify the balance between the sympathetic and parasympathetic nerves (Shaffer and Ginsberg, 2017).

All the aforementioned data processing and analysis steps were completed using the RHRV package in R software (Rodríguez-Liñares et al., 2011).

In addition, an echocardiogram and an electrocardiogram were also performed on each patient at baseline.

### 2.6 Grouping

SDNN, RMSSD, and the LF/HF ratio were selected as the core indicators for grouping. In this study, “autonomic abnormality” was used for research definition, grouping, and objective quantitative judgment based on HRV parameters, whereas “autonomic dysfunction” was used to describe clinical manifestations, symptoms, and pathological conditions, and was not used for baseline grouping or prognostic analysis. Patients with abnormalities in at least one indicator were assigned to the HRV-abnormal group (i.e., the autonomic abnormality group), while those with normal results in all three indicators were assigned to the HRV-normal group.

The cut-off values for normal and abnormal HRV indices were determined based on the gold standard guidelines for HRV measurement and interpretation (Task Force of the European Society of Cardiology and the North American Society of Pacing and Electrophysiology, 1996), and age-corrected for older patients aged ≥60 years with reference to the normative data of 5-min resting short-term HRV and age-related physiological characteristics (Shaffer and Ginsberg, 2017), combined with the core physiological mechanisms underlying the association between HRV and autonomic nervous function (McCraty and Shaffer, 2015). The normal reference ranges were defined as follows: SDNN 30–80 ms, RMSSD 10–30 ms, and LF/HF 0.8–2.5. An index value below the lower limit (for SDNN and RMSSD) or outside the range (for LF/HF) was considered abnormal. The age correction was performed by lowering the baseline HRV ranges of healthy adults by 30–50% in accordance with the physiological law of age-related decline in autonomic nervous function in the elderly, which is suitable for the clinical and physiological characteristics of older patients with ACI.

This grouping design was based on the fact that the three indicators (SDNN, RMSSD, and LF/HF) systematically and non-redundantly cover the three core dimensions of autonomic nervous function assessment, namely global regulation, parasympathetic activity, and sympathetic-parasympathetic balance. Collectively, these three indicators comprehensively reflect the overall regulatory state and functional characteristics of the autonomic nervous system. Meanwhile, an abnormality in any one of these three indicators can independently indicate a regulatory imbalance or functional impairment in a specific dimension of the autonomic nervous system, and each indicator has independent clinical significance for suggesting autonomic dysfunction. Therefore, SDNN, RMSSD, and LF/HF meet the requirements for dimensional representativeness and clinical discriminability of core indicators for grouping. In contrast, pNN50 and HF, along with RMSSD, all target the dimension of parasympathetic function assessment and exhibit obvious functional overlap; the core evaluative value of LF has been integrated into the quantitative analysis of sympathetic-parasympathetic balance by the LF/HF ratio, and its separate inclusion has no additional dimensional significance. For these reasons, these three indicators were not used as core grouping indicators and were only applied for the descriptive analysis of autonomic nervous function between groups.

### 2.7 Psychocognitive and functional assessment

At baseline and at 1-year follow-up, posttraumatic growth, cognitive function, and functional ability (representing activities of daily living) were assessed.

Posttraumatic growth was evaluated using the Posttraumatic Growth Inventory (PTGI) and Connor-Davidson Resilience Scale (CD-RISC). The PTGI assesses positive psychological changes after trauma, comprising 21 items across 5 domains with a 6-point scale (0 = no change, 5 = extreme change), yielding a total score of 0–105 (higher scores indicate greater posttraumatic growth) (Tedeschi et al., 2017). The CD-RISC measures psychological resilience to stress, containing 25 items with a 5-point scale (0 = never true, 4 = always true), with total scores ranging 0–100 (higher scores reflect stronger resilience) (Kuiper et al., 2019). In this study, the scores of both scales were dichotomized based on the median scores of the study population, with scores ≥ the median indicating a higher level of posttraumatic growth or psychological resilience, and scores < the median indicating a lower level.

Cognitive function was assessed via the Montreal Cognitive Assessment (MoCA) and Addenbrooke’s cognitive examination-III (ACE-III). The MoCA, a screening tool for mild cognitive impairment, includes 11 cognitive domain items scored 0–30; scores ≤26 suggest cognitive impairment (with a + 1 adjustment for ≤12 years of education) (Nasreddine et al., 2005). The ACE-III is another tool for assessing cognitive function. Comprising 21 items with a total score of 100, it covers five cognitive domains: attention/orientation, memory, language, visuospatial ability, and executive function. Its scoring depends on the examinee’s education level: scores ≤72 (primary education or below), ≤78 (secondary education), or ≤80 (college education or above) may indicate mild cognitive impairment (Bruno and Schurmann Vignaga, 2019).

Functional ability was assessed using the Modified Barthel Index (MBI) and Functional Independence Measure (FIM). The MBI evaluates activities of daily living (ADL), including 10 items such as eating and dressing, with a total score ranging from 0 to 100; a score of ≥60 indicates basic self-care in daily life, while a score of <60 indicates dependence, with higher scores reflecting greater self-care ability (Wang et al., 2023). The FIM is a comprehensive functional assessment tool covering 6 domains including self-care and mobility, with each item rated on a 7-point scale (1 = total dependence, 7 = complete independence) and a total score ranging from 18 to 126; a score of ≥90 indicates basic functional independence, while a score of <90 indicates functional dependence, with higher scores indicating better functional independence (Ribeiro et al., 2018).

### 2.8 Statistical analysis

Continuous variables were expressed as mean±standard deviation, and intergroup differences were evaluated using independent samples *t*-tests. Categorical variables were presented as frequencies and proportions, with intergroup differences assessed via chi-square tests.

Multivariate logistic regression analyses were performed to compare changes in psychocognitive and functional status (PTGI, CD-RISC, MoCA, ACE-III, MBI, and FIM scores) from baseline to at 1-year follow-up, with baseline scores as the independent variables and 1-year follow-up scores as the dependent variables. Additionally, multivariate logistic regression analyses were conducted to assess the association between baseline autonomic abnormality (HRV abnormality) and psychocognitive and functional status at 1-year follow-up, where baseline autonomic abnormality served as the independent variable and the aforementioned six psychocognitive and functional scores as the dependent variables.

For the purpose of these multivariate analyses, autonomic abnormality was defined according to the baseline HRV grouping status (HRV-normal vs. HRV-abnormal), which was consistent with the grouping strategy applied in the preceding *t*-tests and chi-square tests. All six psychocognitive and functional scores were converted into dichotomous variables for regression analyses: the PTGI and CD-RISC scores were dichotomized based on the median scores of the study population, whereas the MoCA, ACE-III, MBI, and FIM scores were dichotomized in accordance with their established clinical criteria (the gold standard for score stratification in clinical practice).

These multivariate analyses were adjusted for potential confounding factors including demographic data, personal history, disease history, medication history, characteristics of acute cerebral infarction, acute-phase treatment, echocardiogram at baseline, electrocardiogram at baseline, and other cerebral infarction-related treatments during the follow-up.

A *p*-value < 0.05 was considered statistically significant. All analyses were performed using SPSS 27.0.

## 3 Results

### 3.1 Participants

Initially, a total of 465 patients met the aforementioned inclusion criteria. During the follow-up period, however, 39 patients (8.4%) experienced a recurrent acute cerebrovascular event, and an additional 6 patients (1.3%) declined to continue participating in the study due to personal reasons. Consequently, 420 patients (90.3%) completed the study.

In accordance with the above criteria, 113 participants were assigned to the HRV-normal group, and 307 participants were assigned to the HRV-abnormal group.

### 3.2 Basic characteristics of older patients at disease onset

In Table 1, compared with the HRV-normal group, the HRV-abnormal group had a higher proportion of older patients with a smoking history, a drinking history, and a tendency toward a high-salt diet (*p* = 0.028, *p* = 0.020, *p* = 0.047). Additionally, the proportion of older patients with hypertension, coronary heart disease, and obstructive sleep apnea was higher in the HRV-abnormal group (*p* = 0.001, *p* = 0.007, *p* = 0.031). There were no significant differences in demographic data or medication history between the two groups (all *p* > 0.05).

**Table 1:** Basic characteristics of the older patients in the two groups at disease onset.

Variable	HRV-normal group	HRV-abnormal group	*t*/*χ*^2^ value	*p* value
Total (n)	113 (100.0)	307 (100.0)	–	–
Demographic data				
Age (yr)	68.7 ± 5.5	69.1 ± 5.3	0.546	0.585
Gender (n)				
Male	70 (61.9)	171 (55.7)	1.318	0.251
Female	43 (38.1)	136 (44.3)		
Education (n)				
>12 years	31 (27.4)	102 (33.2)	1.280	0.258
≤12 years	82 (72.6)	205 (66.8)		
Personal history				
Smoking history (n)	26 (23.0)	105 (34.2)	4.822	0.028
Drinking history (n)	31 (27.4)	122 (39.7)	5.401	0.020
Physical activity (n)				
<2 h/wk	59 (52.2)	191 (62.2)	3.430	0.064
≥2 h/wk	54 (47.8)	116 (37.8)		
High-fat diet (n)	43 (38.1)	147 (47.9)	3.222	0.073
High-salt diet (n)	57 (50.4)	188 (61.2)	3.960	0.047
Disease history				
Hypertension (n)	62 (54.9)	219 (71.3)	10.117	0.001
Coronary heart disease (n)	23 (20.4)	104 (33.9)	7.160	0.007
Chronic heart failure (n)	7 (6.2)	38 (12.4)	3.301	0.069
Type 2 diabetes (n)	39 (34.5)	128 (41.7)	1.778	0.182
Hyperlipidemia (n)	55 (48.7)	171 (55.7)	1.641	0.200
OSA (n)	21 (18.6)	89 (29.0)	4.627	0.031
Medication history				
β-blockers (n)	23 (20.4)	86 (28.0)	2.521	0.112
CCBs (n)	25 (22.1)	74 (24.1)	0.180	0.672
ACEIs/ARBs (n)	35 (31.0)	110 (35.8)	0.862	0.353
Diuretics (n)	15 (13.3)	49 (16.0)	0.462	0.497
Sulfonylureas (n)	11 (9.7)	36 (11.7)	0.330	0.566

HRV, Heart rate variability; OSA, Obstructive sleep apnea; CCB, Calcium channel blocker; ACEI, Angiotensin-converting enzyme inhibitor; ARB, Angiotensin II receptor blocker.

Continuous variables were presented as mean ± standard deviation, and categorical variables were expressed as frequencies and proportions. A *p*-value < 0.05 indicated statistical significance.

### 3.3 Infarction characteristics and acute-phase treatment

In Table 2, compared with the HRV-normal group, older patients in the HRV-abnormal group had a higher NIHSS score before treatment (*p* = 0.027), and also had a higher proportion of mTICI grade 0/1 after interventional therapy (*p* = 0.039). There were no significant differences between the two groups in terms of infarct location, infarct volume, time from onset to interventional therapy, interventional therapy method, interventional therapy complications, and other therapies (all *p* > 0.05).

**Table 2:** Infarction characteristics and acute-phase treatment in the older patients of the two groups.

Variable	HRV-normal group	HRV-abnormal group	*t*/*χ*^2^ value	*p* value
Total (n)	113 (100.0)	307 (100.0)	–	–
Pre-treatment				
NIHSS	11.3 ± 4.5	12.4 ± 4.7	2.225	0.027
Infarct location (n)				
Anterior circulation	77 (68.1)	187 (60.9)	1.849	0.174
Posterior circulation	27 (23.9)	82 (26.7)	0.341	0.559
Serial occlusion	9 (8.0)	38 (12.4)	1.619	0.203
Infarct volume≥50 mL (n)	17 (15.0)	71 (23.1)	3.258	0.071
Intervention therapy				
Time O2IT (h)	5.1 ± 1.6	4.9 ± 1.6	1.377	0.169
Therapy method (n)				
MT	45 (39.8)	132 (43.0)	0.341	0.559
IAT	32 (28.3)	98 (31.9)	0.502	0.479
BT	22 (19.5)	68 (22.1)	0.353	0.553
PTA	15 (13.3)	45 (14.7)	0.129	0.719
SI	12 (10.6)	35 (11.4)	0.051	0.822
mTICI grade (n)				
Grade 0/1	13 (11.5)	62 (20.2)	4.253	0.039
Grade 2a/2b/3	100 (88.5)	245 (79.8)		
Complication (n)				
ICH	6 (5.3)	31 (10.1)	2.357	0.125
DE	8 (7.1)	37 (12.1)	2.135	0.144
CV	5 (4.4)	28 (9.1)	2.516	0.113
Other therapy				
IVT (n)	24 (21.2)	82 (26.7)	1.310	0.252
Antiplatelet (n)	97 (85.8)	283 (92.2)	3.855	0.050
Statins (n)	89 (78.8)	264 (86.0)	3.222	0.073
Neuroprotectants (n)	65 (57.5)	208 (67.8)	3.800	0.051
Dehydration therapy (n)	47 (41.6)	148 (48.2)	1.453	0.228

HRV, Heart rate variability; NIHSS, National institute of health stroke scale; Time O2IT, Time from onset to interventional therapy; MT, Mechanical thrombectomy; IAT, Intra - arterial thrombolysis; BT, Bridging therapy; PTA, Percutaneous transluminal angioplasty; SI, Stent implantation; mTICI, Modified thrombolysis in cerebral infarction; ICH, Intracranial hemorrhage; DE, Distal embolization; CV, Cerebral vasospasm; IVT, Intravenous thrombolysis.

Continuous variables were presented as mean ± standard deviation, and categorical variables were expressed as frequencies and proportions. A *p*-value < 0.05 indicated statistical significance.

### 3.4 Baseline heart rate variability and other cardiac indicators

In Table 3, compared with the HRV-normal group, the HRV-abnormal group had lower levels of RMSSD, SDNN, and HF (*p* < 0.001, p < 0.001, *p* = 0.002), as well as higher levels of LF and LF/HF ratio (*p* = 0.011, p < 0.001). There were no significant differences in echocardiogram and electrocardiogram (ECG) parameters between the two groups (all *p* > 0.05). Notably, the frequency of some ECG parameters was too low in both groups, so no statistical analysis was performed for these parameters.

**Table 3:** Baseline heart rate variability and other cardiac indicators in the older patients of the two groups.

Variable	HRV-normal group	HRV-abnormal group	*t*/*χ*^2^ value	*p* value
Total (n)	113 (100.0)	307 (100.0)	–	–
Heart rate variability				
RMSSD (ms)	22.4 ± 6.4	15.2 ± 10.3	6.923	<0.001
SDNN (ms)	97.8 ± 26.8	65.1 ± 42.2	7.673	<0.001
pNN50 (%)	10.5 ± 5.2	10.0 ± 5.7	0.882	0.378
HF (ms^2^)	202.2 ± 88.5	170.0 ± 96.9	3.090	0.002
LF (ms^2^)	246.8 ± 145.4	322.9 ± 302.6	2.565	0.011
LF/HF ratio	1.2 ± 0.4	1.9 ± 1.2	6.013	<0.001
Echocardiogram				
LVEF (%)	56.8 ± 5.7	55.9 ± 7.3	1.119	0.264
E/A ratio	1.2 ± 0.2	1.1 ± 0.3	1.580	0.115
LVEDD (mm)	48.6 ± 4.5	49.3 ± 5.0	1.366	0.173
RVEDD (mm)	38.8 ± 4.1	39.3 ± 5.3	0.973	0.331
Electrocardiogram				
Heart rate (bpm)	69.8 ± 5.5	71.4 ± 9.3	1.744	0.082
ST-T changes (n)	1 (0.9)	2 (0.7)	–	–
T-wave abnormalities (n)	1 (0.9)	2 (0.7)	–	–
AVB grade I-II (n)	0 (0.0)	1 (0.3)	–	–
IVCB (n)	0 (0.0)	1 (0.3)	–	–
QTc (ms)	408.9 ± 26.4	412.4 ± 32.1	1.024	0.306

HRV, Heart rate variability; RMSSD, Root mean square of successive differences; SDNN, Standard deviation of normal-to-normal intervals; pNN50, Percentage of successive normal-to-normal intervals differing by more than 50 ms; HF, High frequency; LF, Low frequency; LVEF, Left ventricular ejection fraction; LVEDD, Left ventricular end-diastolic diameter; RVEDD, Right ventricular end-diastolic diameter; AVB, Atrioventricular block; IVCB, Intraventricular conduction block; QTc, Corrected QT interval.

Continuous variables were presented as mean ± standard deviation, and categorical variables were expressed as frequencies and proportions. A *p*-value < 0.05 indicated statistical significance.

### 3.5 Cerebral infarction-related treatments during 1-year follow-up

In Table 4, there were no significant differences between the two groups in the treatments administered for cerebral infarction during the follow-up period, including antiplatelet agents, statins, rehabilitation therapy, neuroprotectants, cerebral circulation and metabolism agents, traditional Chinese medicine therapy, and anticoagulants (all *p* > 0.05).

**Table 4:** Cerebral infarction-related treatments in the older patients of the two groups during 1-year follow-up.

Variable	HRV-normal group	HRV-abnormal group	*t*/*χ*^2^ value	*p* value
Total (n)	113 (100.0)	307 (100.0)	–	–
Antiplatelet agents (n)	103 (91.2)	291 (94.8)	1.882	0.170
Statins (n)	96 (85.0)	273 (88.9)	1.220	0.269
Rehabilitation therapy (n)	87 (77.0)	251 (81.8)	1.195	0.274
Neuroprotectants (n)	65 (57.5)	201 (65.5)	2.248	0.134
CC&M agents (n)	46 (40.7)	147 (47.9)	1.712	0.191
TCM therapy (n)	28 (24.8)	83 (27.0)	0.216	0.642
Anticoagulants (n)	6 (5.3)	21 (6.8)	0.322	0.571

HRV, Heart rate variability; TCM, Traditional Chinese medicine; CC&M, Cerebral circulation & metabolism.

Continuous variables were presented as mean ± standard deviation, and categorical variables were expressed as frequencies and proportions. A *p*-value < 0.05 indicated statistical significance.

### 3.6 Psychocognitive and functional status at baseline and 1-year follow-up

As shown in Table 5, at baseline, there were no significant differences in the six scores of posttraumatic growth, cognitive function, and functional ability between the two groups (all p > 0.05), and the baseline scores were also very close in clinical terms, indicating good comparability of the two groups at the study outset. At 1-year follow-up, compared with the HRV-normal group, the HRV-abnormal group had significantly lower scores in posttraumatic growth (including PTGI and CD-RISC), cognitive function (including MoCA and ACE-III), and functional ability (including MBI and FIM) (all *p* < 0.001).

**Table 5:** Psychocognitive and functional status of the older patients in the two groups at baseline and 1-year follow-up.

Variable	HRV-normal group	HRV-abnormal group	*t*/*χ*^2^ value	*p* value
Total (n)	113 (100.0)	307 (100.0)	–	–
At baseline				
Posttraumatic growth				
PTGI	31.7 ± 2.1	31.6 ± 1.9	0.734	0.463
CD-RISC	65.2 ± 3.1	64.9 ± 2.9	0.887	0.376
Cognitive function				
MoCA	24.3 ± 1.4	24.1 ± 1.2	1.033	0.302
ACE-III	83.0 ± 3.7	82.6 ± 4.3	0.920	0.358
Functional ability				
MBI	70.0 ± 2.7	69.7 ± 3.3	0.892	0.373
FIM	76.1 ± 2.9	75.8 ± 3.0	0.987	0.324
At 1-year follow-up				
Posttraumatic growth				
PTGI	33.3 ± 2.5	31.4 ± 2.1	7.850	<0.001
CD-RISC	68.8 ± 3.7	64.7 ± 3.6	10.352	<0.001
Cognitive function				
MoCA	25.7 ± 1.6	23.9 ± 1.5	10.649	<0.001
ACE-III	85.8 ± 4.1	82.5 ± 3.3	8.690	<0.001
Functional ability				
MBI	73.6 ± 3.3	69.6 ± 3.7	10.330	<0.001
FIM	78.1 ± 3.3	75.5 ± 3.5	6.840	<0.001

HRV, Heart rate variability; PTGI, Posttraumatic growth inventory; CD-RISC, Connor-davidson resilience scale; MoCA, Montreal cognitive assessment; ACE-III, Addenbrooke’s cognitive examination-III; MBI, Modified barthel index; FIM, Functional independence measure.

Continuous variables were presented as mean ± standard deviation, and categorical variables were expressed as frequencies and proportions. A *p*-value < 0.05 indicated statistical significance.

### 3.7 Multivariate logistic regression analysis for psychocognitive and functional status changes from baseline to 1-year follow-up

As shown in Table 6, after adjusting for the potential confounding factors mentioned above, multivariate logistic regression analysis revealed that in the HRV-normal group, the ORs for all six scores (PTGI, CD-RISC, MoCA, ACE-III, MBI, FIM) were greater than 1 (ranging from 1.201 to 1.885), with all *p* < 0.001—indicating that the probability of achieving favorable performance in these psychocognitive and functional scores at 1-year follow-up was significantly increased by 20.1 to 88.5% compared with baseline. In the HRV-abnormal group, by contrast, the ORs for all six scores were less than 1 (ranging from 0.896 to 0.993), with all *p* > 0.05, suggesting that the probability of favorable performance in these scores at 1-year follow-up did not increase relative to baseline and even tended to decrease, with no statistically significant differences observed.

**Table 6:** Multivariate logistic regression analysis for psychocognitive and functional status changes from baseline to 1-year follow-up in the older patients.

Variable	*p* value	OR (95%CI)
HRV-normal group (Independent vs. dependent variable)		
PTGI (at baseline vs. at 1-year follow-up)	<0.001	1.346 (1.189 ~ 1.524)
CD-RISC (at baseline vs. at 1-year follow-up)	<0.001	1.349 (1.230 ~ 1.480)
MoCA (at baseline vs. at 1-year follow-up)	<0.001	1.885 (1.526 ~ 2.329)
ACE-III (at baseline vs. at 1-year follow-up)	<0.001	1.201 (1.118 ~ 1.291)
MBI (at baseline vs. at 1-year follow-up)	<0.001	1.510 (1.340 ~ 1.702)
FIM (at baseline vs. at 1-year follow-up)	<0.001	1.224 (1.118 ~ 1.339)
HRV-abnormal group (Independent vs. dependent variable)		
PTGI (at baseline vs. at 1-year follow-up)	0.278	0.957 (0.885 ~ 1.036)
CD-RISC (at baseline vs. at 1-year follow-up)	0.391	0.979 (0.932 ~ 1.028)
MoCA (at baseline vs. at 1-year follow-up)	0.168	0.896 (0.796 ~ 1.008)
ACE-III (at baseline vs. at 1-year follow-up)	0.750	0.993 (0.953 ~ 1.035)
MBI (at baseline vs. at 1-year follow-up)	0.702	0.991 (0.947 ~ 1.037)
FIM (at baseline vs. at 1-year follow-up)	0.250	0.972 (0.926 ~ 1.020)

HRV, Heart rate variability; PTGI, Posttraumatic growth inventory; CD-RISC, Connor-davidson resilience scale; MoCA, Montreal cognitive assessment; ACE-III, Addenbrooke’s cognitive examination-III; MBI, Modified barthel index; FIM, Functional independence measure; OR, Odds ratio; 95%CI, 95% Confidence interval.

Multivariate logistic regression was performed, with the following factors adjusted: demographic data, personal history, disease history, medication history, characteristics of acute cerebral infarction, acute-phase treatment, echocardiogram at baseline, electrocardiogram at baseline, and other cerebral infarction-related treatments during the follow-up.

### 3.8 Multivariate logistic regression analysis for the association of baseline HRV abnormality with psychocognitive and functional status at 1-year follow-up

As shown in Table 7, after adjusting for the same set of confounding factors mentioned above, multivariate logistic regression analysis demonstrated that baseline HRV abnormality (i.e., autonomic abnormality) was significantly and negatively associated with favorable performance in all six psychocognitive and functional scores at 1-year follow-up (all *p* < 0.001). The ORs for the association between baseline autonomic abnormality (HRV abnormality) and these six scores (PTGI, CD-RISC, MoCA, ACE-III, MBI, FIM) were all less than 1 (ranging from 0.466 to 0.806), indicating that compared with the HRV-normal group, older patients with baseline autonomic abnormality had a 19.4 to 53.4% lower probability of achieving favorable psychocognitive and functional performance at 1-year follow-up.

**Table 7:** Multivariate logistic regression analysis for the association of baseline HRV abnormality with psychocognitive and functional status at 1-year follow-up in the older patients.

Independent vs. dependent variable	*p* value	OR (95%CI)
HRV-abnormal at baseline vs. PTGI at 1-year follow-up	<0.001	0.686 (0.616 ~ 0.763)
HRV-abnormal at baseline vs. CD-RISC at 1-year follow-up	<0.001	0.733 (0.681 ~ 0.790)
HRV-abnormal at baseline vs. MoCA at 1-year follow-up	<0.001	0.466 (0.389 ~ 0.559)
HRV-abnormal at baseline vs. ACE-III at 1-year follow-up	<0.001	0.770 (0.718 ~ 0.825)
HRV-abnormal at baseline vs. MBI at 1-year follow-up	<0.001	0.719 (0.664 ~ 0.778)
HRV-abnormal at baseline vs. FIM at 1-year follow-up	<0.001	0.806 (0.753 ~ 0.863)

HRV, Heart rate variability; PTGI, Posttraumatic growth inventory; CD-RISC, Connor-davidson resilience scale; MoCA, Montreal cognitive assessment; ACE-III, Addenbrooke’s cognitive examination-III; MBI, Modified barthel index; FIM, Functional independence measure; OR, Odds ratio; 95%CI, 95% Confidence interval.

Multivariate logistic regression was performed, with the following factors adjusted: demographic data, personal history, disease history, medication history, characteristics of acute cerebral infarction, acute-phase treatment, echocardiogram at baseline, electrocardiogram at baseline, and other cerebral infarction-related treatments during the follow-up.

## 4 Discussion

Exploring the significance of baseline autonomic abnormality (assessed by HRV) for long-term psychocognitive and functional prognoses in older patients with acute cerebral infarction can provide preliminary objective evidence for early risk stratification and recovery trend prediction. It addresses the oversight of the association with autonomic nervous function in traditional assessments and, to a certain extent, highlights its potential value as a reversible intervention target, offering important references for developing targeted rehabilitation protocols and improving patients’ long-term quality of life.

The reasons why this study focuses on the older population are as follows: (1) The older population inherently exhibits age-related autonomic dysfunction to varying degrees (Sato et al., 2021); when superimposed with ACI-induced injury, this dysfunction becomes more prominent and exerts a more significant impact on prognosis. (2) Older patients are a high-risk group for psychocognitive impairments and delayed functional recovery after ACI. It is therefore necessary to focus on exploring the factors influencing their prognosis, thereby formulating more precise rehabilitation strategies. Notably, stroke in young adults has been increasingly prevalent in recent years and is accompanied by a much higher morbidity rate, which highlights that the inclusion of young stroke patients in such prognostic studies of autonomic dysfunction is equally important and worthy of further investigation.

It is well established that the location and size of the infarct are key determinants of psychocognitive and functional outcomes following ACI. However, these factors are irreversible and thus difficult to target for clinical prognostic intervention, rendering them of limited practical value in optimizing patient prognosis management. Therefore, there is an urgent need in the medical field to identify additional modifiable and reversible prognostic factors that can provide new entry points for developing targeted rehabilitation strategies and improving patients’ long-term outcomes. As a core non-invasive indicator for assessing autonomic nervous function, HRV reflects the dynamically adjustable state of autonomic regulation, which precisely meets this clinical demand—representing the core academic value and clinical significance of the present study.

The results of this study showed that: in the longitudinal comparison, older patients with ACI who had normal baseline HRV exhibited significant improvements in posttraumatic growth, cognitive function, and functional ability during the follow-up period, with increased scores and a higher probability of favorable performance; in contrast, those with abnormal baseline HRV (i.e., baseline autonomic abnormality) showed no positive changes in these three functional domains. In the cross-sectional comparison, at 1-year follow-up, patients in the HRV-abnormal group had significantly lower scores in the three aforementioned functions and a lower probability of favorable performance compared with the HRV-normal group. These results confirm that HRV is associated with the psychocognitive and functional prognoses of this population, and abnormal baseline HRV (i.e., baseline autonomic abnormality) may be unfavorable to the improvement of their prognoses.

This study controlled confounding factors through two approaches: strict control of participant inclusion criteria and the use of multivariate logistic regression analysis. The inclusion criteria excluded interfering conditions such as cognitive impairment, specific arrhythmias, and autonomic nervous system-related diseases (which may cause autonomic dysfunction), and defined constraints including the time from ACI onset to hospital admission and NIHSS scores, thereby reducing baseline differences. Additionally, multivariate analysis further adjusted for potential confounding factors such as demographic data, personal history, disease history, medication history, ACI characteristics, and treatment regimens. These measures help minimize interference and ensure the independence and reliability of the results.

The assessment of posttraumatic growth, cognitive function, and functional ability is highly subjective, which poses a certain challenge to the stability of the study results. To address this issue, the present study employed two mainstream scales to evaluate each of these functional domains. The results showed that, for each function, the assessment outcomes of the two scales were consistent and mutually corroborative. This alleviated the potential bias caused by subjectivity to a certain extent and enhanced the reliability of the study conclusions.

To evaluate the adequacy of the sample size, this study conducted a *post hoc* test using GPower 3.1.9.7 software with the point-biserial correlation model, based on the results of comparing PTGI scores between the two groups at 1-year follow-up in Table 6. With an effect size set at 0.22, a significance level (*α*) of 0.05, and a total sample size of 420, the results showed that the achieved power reached 0.996, which was much higher than the conventional sufficient threshold, suggesting that the statistical power of this study was adequate (Brydges, 2019).

The potential mechanisms by which baseline autonomic dysregulation (assessed via HRV, i.e., baseline autonomic abnormality) affects the long-term posttraumatic growth, cognitive function, and functional ability of older patients with acute cerebral infarction are as follows:

From the perspective of neural pathway regulation, the autonomic nervous system has extensive bidirectional projections with the central nervous system. The brainstem-prefrontal cortex-limbic system pathway, as a core neural circuit integrating autonomic regulation, cognition, emotion, and stress responses (Melo et al., 2021), is capable of transmitting signals of peripheral autonomic imbalance to the central nervous system, thereby synchronously influencing multidimensional prognostic indicators. It is highly likely the key mediator through which HRV abnormalities (i.e., autonomic abnormality) exert their effects.

Specifically, HRV abnormalities (i.e., autonomic abnormality) in the present study are mainly characterized by excessive sympathetic activation and parasympathetic inhibition. This imbalance first acts on critical brainstem nuclei: On one hand, the locus coeruleus, the central hub of the sympathetic nervous system, becomes overactivated and releases large amounts of norepinephrine. This not only continuously exacerbates neuroinflammatory responses in the brain and inhibits neuroplasticity (Theoharides et al., 2024) but also affects the cognitive control functions of the prefrontal cortex (e.g., regulation of attention and executive function) via the locus coeruleus-prefrontal cortex projection pathway, while hindering the repair and functional maintenance of the hippocampus (the core region for memory encoding and retrieval) (Finke and Schächinger, 2020). On the other hand, dysfunction of the brainstem nucleus tractus solitarius, the central regulator of the parasympathetic nervous system, impairs its ability to effectively filter and buffer stress signals, leading to overactivation of the amygdala (the emotional center within the limbic system). Through the nucleus tractus solitarius-amygdala pathway, this overactivation exacerbates negative emotions such as anxiety and depression, reduces patients’ psychological adaptability to disease-related trauma, and thereby inhibits the development of posttraumatic growth (Blase et al., 2021).

In addition, the autonomic nervous system regulates limb movement via spinal motor neurons and maintains indirect connections with the motor cortex. Its dysfunction impairs the coordination and flexibility of limb movements, reduces patients’ participation in daily activities, and delays the recovery of functional ability. In contrast, older patients with normal baseline HRV can maintain the balance between the sympathetic and parasympathetic nervous systems, stabilize the cognitive regulatory function of the locus coeruleus-prefrontal cortex pathway, preserve the emotional regulatory role of the nucleus tractus solitarius-amygdala pathway, and ensure normal neural innervation of the motor system—collectively laying a unified neurophysiological foundation for the improvement of long-term posttraumatic growth, cognitive function, and functional ability.

Although older patients with definite autonomic nervous system-related disorders (which may cause autonomic dysfunction) were excluded per our enrollment criteria, some participants with occult pre-existing autonomic dysfunction prior to acute cerebral infarction may still have been included in the study. This may increase the population heterogeneity of the HRV-abnormal group (i.e., the autonomic abnormality group), overlook the impacts of underlying comorbidities or living conditions, and impede the differentiation of prognostic effects of autonomic dysfunction with distinct etiologies. However, the core objective of this study was to investigate the effect of short-term autonomic function after acute cerebral infarction on long-term psychocognitive and functional outcomes, without focusing on the etiology of autonomic dysfunction. From a clinical perspective, not excluding such patients does not exert a directional impact on the clinical translation of our study results, as improving autonomic dysfunction and its associated underlying conditions is inherently beneficial to prognostic recovery in patients with stroke. This study limitation thus provides a potential entry point for subsequent stratified research on autonomic dysfunction with different etiologies.

This study has several limitations. First, although all common confounding variables were adjusted as comprehensively as possible, residual confounding factors may still exist, which is mainly restricted by the complexity of clinical data collection and the scope of the current statistical model. Second, in terms of the specificity of HRV measurement, HRV can reflect the overall tone and balance of the autonomic nervous system, but cannot accurately locate the lesion site in the central (e.g., brainstem, insula) or peripheral nervous system. In addition, although respiration was controlled during measurement, transient emotional state and other interindividual differences before testing may still cause slight fluctuations in HRV parameters. Third, all older patients in this study were derived from a single center and received interventional therapy, which may limit the extrapolation and generalization of the results to other acute cerebral infarction populations, such as those receiving conservative treatment or patients from other medical centers and regions. Fourth, this study did not conduct in-depth mechanistic exploration. Because the current research focused on prognostic validation, the underlying biological and pathophysiological pathways were not elucidated. Future studies with larger sample sizes, multicenter designs, and strict quality control of HRV detection are warranted to minimize confounding bias. Further mechanistic studies combined with molecular biology and imaging techniques are also needed to verify and refine the present findings.

In conclusion, this study preliminarily confirms that early autonomic dysfunction in older patients with acute cerebral infarction may affect their long-term psychocognitive and functional prognoses, providing preliminary evidence for exploring prognostic risk factors in this population. Our findings suggest that incorporating early autonomic function indicators (such as heart rate variability, which is used to assess autonomic abnormality) into the clinical prognostic evaluation system can help identify high-risk individuals at an early stage. More importantly, heart rate variability may serve as a potentially reversible intervention target. Future research is warranted to further explore whether targeted autonomic interventions (e.g., heart rate variability biofeedback, breathing exercises, and related medications) can improve heart rate variability (i.e., alleviate autonomic abnormality) in older patients with acute cerebral infarction and thereby promote their psychocognitive functional recovery, so as to provide more translationally valuable clinical strategies for implementing individualized interventions and improving the long-term quality of life of these patients.

## Data Availability

The raw data supporting the conclusions of this article will be made available by the authors, without undue reservation.

## References

[ref1] Barkhudaryan A. Doehner W. Jauert N. (2025). Autonomic dysfunction after stroke: an overview of recent clinical evidence and perspectives on therapeutic management. Clin. Auton. Res. 35, 553–563. doi: 10.1007/s10286-025-01120-0, 40131648 PMC12325444

[ref2] Blase K. Vermetten E. Lehrer P. Gevirtz R. (2021). Neurophysiological approach by self-control of your stress-related autonomic nervous system with depression, stress and anxiety patients. Int. J. Environ. Res. Public Health 18:3329. doi: 10.3390/ijerph18073329, 33804817 PMC8036915

[ref3] Bruno D. Schurmann Vignaga S. (2019). Addenbrooke's cognitive examination III in the diagnosis of dementia: a critical review. Neuropsychiatr. Dis. Treat. 15, 441–447. doi: 10.2147/NDT.S151253, 30858702 PMC6387595

[ref4] Brydges C. R. (2019). Effect size guidelines, sample size calculations, and statistical power in gerontology. Innov. Aging 3:igz036. doi: 10.1093/geroni/igz036 31528719 PMC6736231

[ref5] Calhoun L. G. Cann A. Tedeschi R. G. McMillan J. (2000). A correlational test of the relationship between posttraumatic growth, religion, and cognitive processing. J. Trauma. Stress. 13, 521–527. doi: 10.1023/A:1007745627077, 10948491

[ref6] Finke J. B. Schächinger H. (2020). Central sympathetic nervous system effects on cognitive-motor performance. Exp. Psychol. 67, 77–87. doi: 10.1027/1618-3169/a000475, 32729404

[ref7] Gangstad B. Norman P. Barton J. (2009). Cognitive processing and posttraumatic growth after stroke. Rehabil. Psychol. 54, 69–75. doi: 10.1037/a0014639, 19618705

[ref8] Gu P. Ding Y. Ruchi M. Feng J. Fan H. Fayyaz A. . (2024). Post-stroke dizziness, depression and anxiety. Neurol. Res. 46, 466–478. doi: 10.1080/01616412.2024.2328490, 38488118

[ref9] Hourani L. L. Davila M. I. Morgan J. Meleth S. Ramirez D. Lewis G. . (2020). Mental health, stress, and resilience correlates of heart rate variability among military reservists, guardsmen, and first responders. Physiol. Behav. 214:112734. doi: 10.1016/j.physbeh.2019.112734, 31722190

[ref10] Karaman İ. G. Y. Kaçar C. Y. Dirik G. Ersan İ. Demir N. Mert G. Ö. (2025). Posttraumatic stress, posttraumatic growth, and heart rate variability among breast Cancer survivors. Noro Psikiyatr. Ars. 62, 150–155. doi: 10.29399/npa.28713, 40583954 PMC12205382

[ref11] Klass M. Rogers K. Dorstyn D. Kneebone I. I. (2025). Posttraumatic growth after stroke: a systematic review and meta-regression. Disabil. Rehabil., 1–12. doi: 10.1080/09638288.2025.2540070, 40762138

[ref12] Kuiper H. van Leeuwen C. C. M. Stolwijk-Swüste J. M. Post M. W. M. (2019). Measuring resilience with the Connor-Davidson resilience scale (CD-RISC): which version to choose? Spinal Cord 57, 360–366. doi: 10.1038/s41393-019-0240-1, 30670770

[ref13] Lago S. de Beukelaar T. T. Casetta I. Arcara G. Mantini D. (2025). Heart rate variability and autonomic dysfunction after stroke: prognostic markers for recovery. Biomedicine 13:1659. doi: 10.3390/biomedicines13071659, 40722730 PMC12292726

[ref14] Lombardi F. Huikuri H. Schmidt G. Malik M. e-Rhythm Study Group of European Heart Rhythm Association (2018). Short-term heart rate variability: easy to measure, difficult to interpret. Heart Rhythm. 15, 1559–1560. doi: 10.1016/j.hrthm.2018.05.023, 29803853

[ref15] McCraty R. Shaffer F. (2015). Heart rate variability: new perspectives on physiological mechanisms, assessment of self-regulatory capacity, and health risk. Glob Adv Health Med 4, 46–61. doi: 10.7453/gahmj.2014.073, 25694852 PMC4311559

[ref16] Melo H. M. de Carvalho C. R. Hoeller A. A. Marques J. L. B. Linhares M. N. Lopes M. W. . (2021). AMPAr GluA1 phosphorylation at serine 845 in limbic system is associated with cardiac autonomic tone. Mol. Neurobiol. 58, 1859–1870. doi: 10.1007/s12035-020-02272-y, 33404979

[ref17] Mendelson S. J. Prabhakaran S. (2021). Diagnosis and management of transient ischemic attack and acute ischemic stroke: a review. JAMA 325, 1088–1098. doi: 10.1001/jama.2020.26867, 33724327

[ref18] Morgan A. E. Mc Auley M. T. (2024). Vascular dementia: from pathobiology to emerging perspectives. Ageing Res. Rev. 96:102278. doi: 10.1016/j.arr.2024.102278, 38513772

[ref19] Nasreddine Z. S. Phillips N. A. Bédirian V. Charbonneau S. Whitehead V. Collin I. . (2005). The Montreal cognitive assessment, MoCA: a brief screening tool for mild cognitive impairment. J. Am. Geriatr. Soc. 53, 695–699. doi: 10.1111/j.1532-5415.2005.53221.x, 15817019

[ref20] Peng Z. Y. Wan L. H. (2018). Posttraumatic growth of stroke survivors and its correlation with rumination and social support. J. Neurosci. Nurs. 50, 252–257. doi: 10.1097/JNN.0000000000000371, 29985279

[ref21] Ribeiro D. K. M. N. Lenardt M. H. Lourenço T. M. Betiolli S. E. Seima M. D. Guimarães C. A. (2018). The use of the functional independence measure in elderly. Rev. Gaucha Enferm. 38:e66496. 29933424 10.1590/1983-1447.2017.04.66496

[ref22] Rodríguez-Liñares L. Méndez A. J. Lado M. J. Olivieri D. N. Vila X. A. Gómez-Conde I. (2011). An open source tool for heart rate variability spectral analysis. Comput. Methods Prog. Biomed. 103, 39–50. doi: 10.1016/j.cmpb.2010.05.012 20674067

[ref23] Sato M. Betriana F. Tanioka R. Osaka K. Tanioka T. Schoenhofer S. (2021). Balance of autonomic nervous activity, exercise, and sleep status in older adults: a review of the literature. Int. J. Environ. Res. Public Health 18:12896. doi: 10.3390/ijerph182412896, 34948506 PMC8701130

[ref24] Shaffer F. Ginsberg J. P. (2017). An overview of heart rate variability metrics and norms. Front. Public Health 5:258. doi: 10.3389/fpubh.2017.00258, 29034226 PMC5624990

[ref25] Sharrief A. Grotta J. C. (2019). Stroke in the elderly. Handb Clin Neurol 167, 393–418. doi: 10.1016/B978-0-12-804766-8.00021-2 31753145

[ref26] Task Force of the European Society of Cardiology and the North American Society of Pacing and Electrophysiology (1996). Heart rate variability: standards of measurement, physiological interpretation and clinical use. Circulation 93, 1043–1065. 8598068

[ref27] Tedeschi R. G. Cann A. Taku K. Senol-Durak E. Calhoun L. G. (2017). The posttraumatic growth inventory: a revision integrating existential and spiritual change. J. Trauma. Stress. 30, 11–18. doi: 10.1002/jts.22155 28099764

[ref28] Theoharides T. C. Twahir A. Kempuraj D. (2024). Mast cells in the autonomic nervous system and potential role in disorders with dysautonomia and neuroinflammation. Ann. Allergy Asthma Immunol. 132, 440–454. doi: 10.1016/j.anai.2023.10.032, 37951572

[ref29] Wang Y. C. Chang P. F. Chen Y. M. Lee Y. C. Huang S. L. Chen M. H. . (2023). Comparison of responsiveness of the Barthel index and modified Barthel index in patients with stroke. Disabil. Rehabil. 45, 1097–1102. doi: 10.1080/09638288.2022.2055166, 35357990

[ref30] Wei C. Han J. Zhang Y. Hannak W. Dai Y. Liu Z. (2017). Affective emotion increases heart rate variability and activates left dorsolateral prefrontal cortex in post-traumatic growth. Sci. Rep. 7:16667. doi: 10.1038/s41598-017-16890-5, 29192184 PMC5709461

[ref31] Xia R. Cai M. Wang Z. Liu X. Pei J. Zaid M. . (2024). Incidence trends and specific risk factors of ischemic heart disease and stroke: an ecological analysis based on the global burden of disease 2019. PLoS Glob Public Health 4:e0003920. doi: 10.1371/journal.pgph.0003920, 39565745 PMC11578511

[ref32] Zhao Y. Hua X. Ren X. Ouyang M. Chen C. Li Y. . (2023). Increasing burden of stroke in China: a systematic review and meta-analysis of prevalence, incidence, mortality, and case fatality. Int. J. Stroke 18, 259–267. doi: 10.1177/17474930221135983, 36274585

